# Cerebroplacental versus Umbilicocerebral Ratio—Analyzing the Predictive Value Regarding Adverse Perinatal Outcomes in Low- and High-Risk Fetuses at Term

**DOI:** 10.3390/medicina59081385

**Published:** 2023-07-28

**Authors:** Florian M. Stumpfe, Andreas Mayr, Michael O. Schneider, Sven Kehl, Frederik Stübs, Sophia Antoniadis, Adriana Titzmann, Constanza A. Pontones, Christian M. Bayer, Matthias W. Beckmann, Florian Faschingbauer

**Affiliations:** 1 Department of Obstetrics and Gynecology, University Hospital of Erlangen, Friedrich-Alexander-Universität Erlangen-Nürnberg, 91054 Erlangen, Germany; florian.stumpfe@fau.de (F.M.S.);; 2 Department of Medical Biometry, Informatics and Epidemiology, Faculty of Medicine, University of Bonn, 53127 Bonn, Germany; 3 WMC Healthcare GmbH, Friedrichstraße 1a, 80801 Munich, Germany

**Keywords:** cerebroplacental ratio, umbilicocerebral ratio, CPR, UCR

## Abstract

*Background and Objectives*: The aim of this study was to investigate the prediction of adverse perinatal outcomes using the cerebroplacental (CPR) and umbilicocerebral (UCR) ratios in different cohorts of singleton pregnancies. *Materials and Methods*: In this retrospective cohort study, we established our own Multiple of Median (MoM) for CPR and UCR. The predictive value for both ratios was studied in the following outcome parameters: emergency cesarean delivery, operative intervention (OI), OI due to fetal distress, 5-min Apgar < 7, admission to neonatal intensive care unit, and composite adverse perinatal outcome. The performance of the ratios was assessed in the following cohorts: total cohort (delivery ≥ 37 + 0 weeks gestation, all birth weight centiles), low-risk cohort (delivery ≥ 37 + 0 weeks gestation, birth weight ≥ 10. centile), prolonged pregnancy cohort (delivery ≥ 41 + 0 weeks gestation, birth weight ≥ 10. centile) and small-for-gestational-age fetuses (delivery ≥ 37 + 0 weeks gestation, birth weight < 10. centile). The underlying reference values for MoM were estimated using quantile regression depending on gestational age. Prediction performance was evaluated using logistic regression models assessing the corresponding Brier score, combining discriminatory power and calibration. *Results*: Overall, 3326 cases were included. Across all cohorts, in the case of a significant association between a studied outcome parameter and CPR, there was an association with UCR, respectively. The Brier score showed only minimal differences for both ratios. *Conclusions*: Our study provides further evidence regarding predictive values of CPR and UCR. The results of our study suggest that reversal of CPR to UCR does not improve the prediction of adverse perinatal outcomes.

## 1. Introduction

Identifying fetuses with an increased risk of a poor perinatal outcome remains a daily challenge. Placental insufficiency does not necessarily lead to low fetal estimated weight or a decline in growth [1]. In recent years, the cerebroplacental ratio (CPR) has become increasingly established as a possible marker for moderate placental insufficiency. Various studies demonstrated that the ratio of the middle cerebral artery (MCA) PI to the umbilical artery (UA) PI is associated with poor perinatal outcomes [2,3]. Especially in fetal growth-restricted (FGR) fetuses, there is good evidence for an association between low CPR and unfavorable outcomes [4,5,6]. On the other hand, previously published studies on small-for-gestational-age (SGA) fetuses and appropriate-for-gestational-age (AGA) fetuses demonstrated heterogeneous results [7,8,9,10]. Despite partially strong associations with several outcome parameters, the prediction of adverse perinatal outcomes remains poor [11]. Due to poor prediction using the CPR, it was proposed to inverse the ratio into the umbilicocerebral ratio (UCR). The UCR was considered to improve the prediction of unfavorable outcomes as this ratio increases with cerebral vasodilatation and increased umbilical resistance, while the CPR approaches zero [12,13]. To date, there is an ongoing debate about the benefit of the UCR compared to the CPR. Thus, our study aimed to compare the predictive value of adverse perinatal outcomes using the CPR and UCR for different groups of fetuses at term. While MoM values for the CPR are already established [14], MoM values are only available to a very limited extent for the UCR. Therefore, we additionally generated MoM values for the CPR and the UCR to compare both ratios.

## 2. Materials and Methods

### 2.1. Study Population

This unicentric, retrospective, cohort study was conducted from July 2016 to March 2019. After searching our obstetrical database (ViewPoint 5.6.26.148; ViewPoint Bildverarbeitung GmbH, Weßling, Germany), all singleton pregnancies with performed Doppler sonography of MCA and UA were identified. In general, in the present study, we analyzed singleton pregnancies with cephalic presentation and planned vaginal delivery. Cases were included in our analyses if Doppler parameters of the umbilical artery and the middle cerebral artery were available. The various study cohorts are discussed in more detail below. Exclusion criteria were cervical opening > 4 cm at the time of ultrasound examination, multiple pregnancies, pregnancies with fetal malformations, intrauterine fetal death, and suspected intrauterine infection.

### 2.2. Doppler Parameters and Ratios

In accordance with our internal hospital standard, a Doppler sonography of MCA and UA were performed at every sonographic examination, and the corresponding pulsatility index (PI) and resistance index (RI) values were stored in our database. The exact technique for Doppler sonography of the various vessels was described in preliminary works [8,9] by our research group. All examinations were carried out with different GE (GE Medical Systems, Zipf, Austria) devices (E10, E8, S8). CPR was calculated as the ratio of MCA PI to UA PI: CPR = MCA PI/UA PI. UCR was calculated as the inverse ratio: UCR = UA PI/MCA PI. 

### 2.3. Study Cohorts

**Figure 1:**
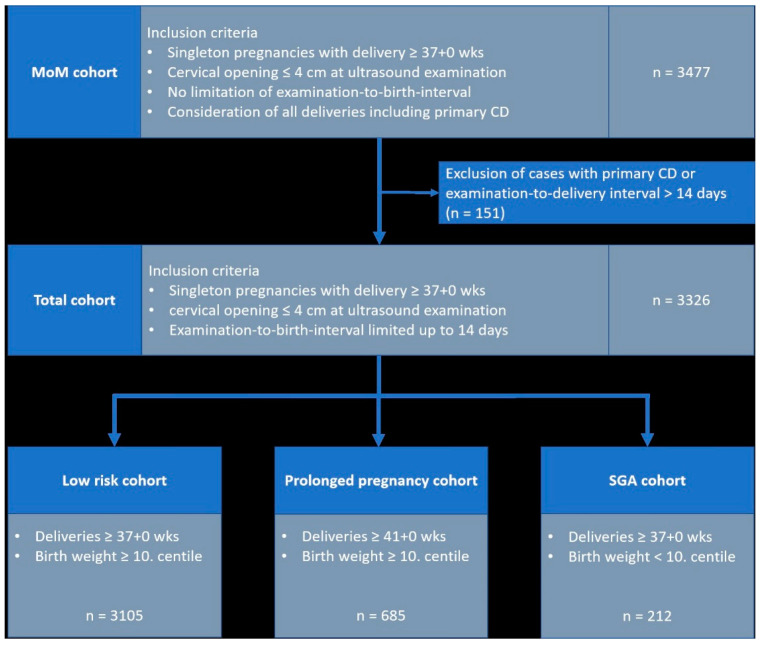
Study cohorts. n number wks, weeks’ gestation; CD, cesarean delivery.

Table 1 and Figure 1 show the different subgroups in this study. Using the MoM cohort, our own Multiples of Median (MoM) values were calculated. The MoM collective consisted of the following cases: all cases with birth from 37 + 0 weeks’ gestation (n = 3477) and cervical opening ≤ 4 cm at examination. The interval between examination and birth was not limited in this cohort. Cases with primary CD were considered.

The evaluation of the association between Doppler parameters and outcome parameters was based on the “Total cohort” (n = 3326). To establish this subgroup, cases with planned cesarean delivery and/or an “examination-to-delivery interval” > 14 days were excluded. In this cohort, all birth centiles were included. In addition to the “Total cohort”, we further conducted a subgroup analysis using the following cohorts:

- *Low-risk cohort*”: all cases with delivery ≥ 37 + 0 weeks gestation, birth weight ≥ 10. centile; n = 3105
- *Prolonged pregnancy cohort*”: all cases with delivery ≥ 41 + 0 weeks gestation and birth weight ≥ 10. centile; n = 685
- *SGA cohort*”: all cases with delivery ≥ 37 + 0 SSW and birth weight < 10. centile; n = 212.

Birth weight centiles were calculated according to the Intergrowth standard [15]. Gestational age was calculated from the last menstrual period and was confirmed or recalculated with crown-rump length measurements from the first trimester (in accordance with the recommendations of the American College of Obstetricians and Gynecologists, ACOG) [16]. In accordance with Bavarian hospital law, patient data collected as part of medical treatment can be used for research and statistics within the hospital. However, the patient data must remain under the hospital’s control. For this reason, we did not require separate informed consent from patients for this retrospective data collection. There were no minors included in this study.

### 2.4. Outcome Definitions

An adverse perinatal outcome was considered in the presence of the following complications: emergency CD, operative intervention (OI) (CD or operative vaginal delivery (OVD)) as well as OI due to fetal distress. In the first step, we studied “emergency CD” as a separate outcome parameter. According to the guidelines of the German Society of Gynecology and Obstetrics [17], an emergency CD was performed in cases of persistent fetal bradycardia or if fetal scalp blood sampling revealed a pH < 7.20. Second, emergency CD is a component of the parameters “operative intervention” and “operative intervention due to fetal distress”. Specifically, the parameter “operative intervention” includes, in addition to vaginal operative deliveries, any secondary cesarean deliveries—i.e., emergency cesarean deliveries and cesarean deliveries indicated during labor—also including CD due to failure to progress in labor. The third outcome parameter, “OI due to fetal distress”, includes OI and CD performed because of “suspected” fetal distress—namely, emergency cesarean sections (under the conditions listed above) and operative interventions due to CTG abnormalities. A pathological CTG trace was defined based on the FIGO consensus guidelines for intrapartum fetal monitoring [18]. Outcome data were collected from the maternity, fetal, and neonatal records in our database. 

An adverse neonatal outcome was assumed if the 5-min Apgar score was <7, if the newborn was admitted to the neonatal intensive care unit (NICU), or in the case of a composite adverse perinatal outcome (CAPO). According to Bligh et al., CAPO summarizes cases with acidosis (UA pH ≤ 7.10) and/or a 5-min Apgar score < 7 and/or NICU admission [19]. 

### 2.5. Statistical Analysis

Descriptive statistics are presented as numbers and percentages for categorical variables (e.g., adverse outcomes) and median values with range (minimum and maximum) for continuous variables. To adjust absolute Doppler values to the corresponding gestational age, we calculated our own MoM values in this study. UA MoMs were calculated as follows: quantile regression models [20] were fitted for the median with the UCR and CPR values as the outcome, and the gestational age in weeks as a linear and squared effect (cf., LIT VA MCA Doppler percentiles) to estimate new reference values. These gestational-age-dependent reference values were then used to compute MoM values for each observation. To assess the predictive value of the CPR and UCR MoM values for adverse perinatal outcomes, separate logistic regression models were computed with the corresponding adverse events as binary responses and the MoM values as single explanatory variables for the different cohorts. For these models, odds ratios (OR) with 95% confidence intervals are reported for the association with the outcome as well as the area under the receiver-operating curve (AUC), representing a measure of discriminatory power. Because discrimination yields identical results for transformations as the inverse ratios represented with the CPR and UCR, we additionally computed the Brier score—taking into account the calibration of the prediction models. In brief, the Brier score can take any value between 0 and 1, where 0 is the best score and 1 is the worst score achievable. The lower the Brier score, the more accurate the prediction. All statistical analyses were performed using the statistical computing environment R 4.1.2.

### 2.6. Ethical Approval

This study was approved by the local ethics committee (September 2016; #275_18 Bc).3. 

## 3. Results

**Figure 2:**
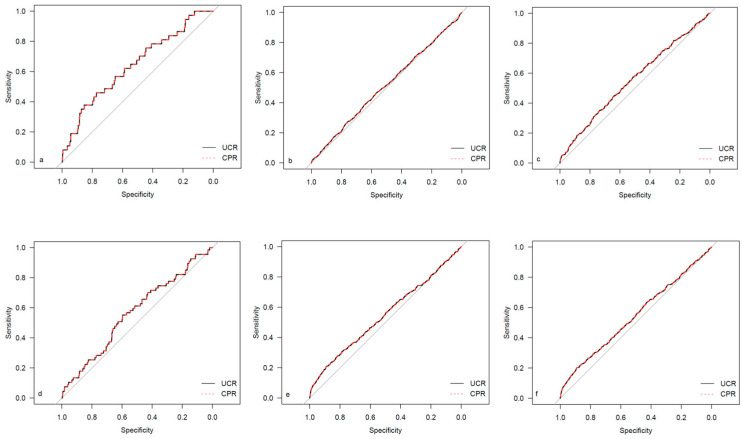
ROC curves for outcome parameters in the total cohort. (**a**) Emergency cesarean; (**b**) operative intervention; (**c**) operative intervention due to fetal distress; (**d**) 5-min Apgar < 7; (**e**) NICU admission; and (**f**) CAPO.

**Figure 3:**
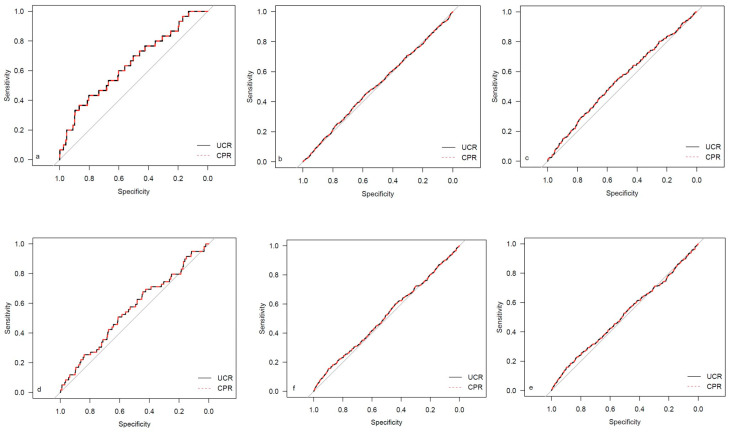
ROC curves for outcome parameters in the low-risk cohort. (**a**) Emergency cesarean; (**b**) operative intervention; (**c**) operative intervention due to fetal distress; (**d**) 5-min Apgar < 7. (**e**) NICU admission; and (**f**) CAPO.

**Figure 4:**
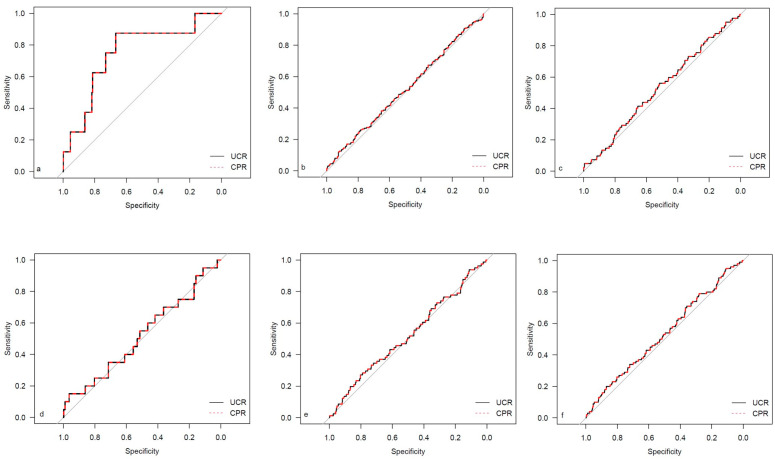
ROC curves for outcome parameters in the prolonged pregnancy cohort. (**a**) Emergency cesarean; (**b**) operative intervention; (**c**) operative intervention due to fetal distress; (**d**) 5-min Apgar < 7; (**e**) NICU admission; and (**f**) CAPO.

**Figure 5:**
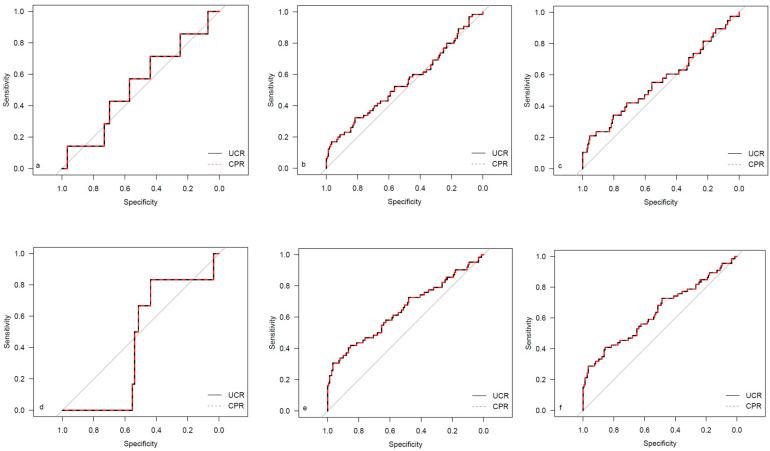
ROC curves for outcome parameters in the SGA cohort. (**a**) Emergency cesarean; (**b**) operative intervention; (**c**) operative intervention due to fetal distress; (**d**) 5-min Apgar < 7; (**e**) NICU admission; and (**f**) CAPO.

The descriptive data for the different study cohorts are shown in Table 1. The univariate regression analysis results for both investigated ratios are presented in Table 2. The corresponding results for the single Doppler parameters (UA MoM and MCA MoM) are shown in Table A1. The results for AUC and Brier scores are given in Table 3 for both ratios. Figure 2, Figure 3, Figure 4 and Figure 5 show the ROC curves for the examined outcome parameters. Table A2 presents the results of the individual Doppler parameters, respectively. Due to the small sample size, the association between Doppler parameters and cases with UA pH ≤ 7.10 was not analyzed.

### 3.1. Emergency Cesarean Delivery

There was a significant association between CPR MoM, UCR MoM, and emergency CD in the total cohort (CPR MoM: OR 0.11; *p* = 0.001; UCR MoM: OR 3.82; *p* = 0.001), the low-risk cohort (CPR MoM: OR 0.10; *p* = 0.002; UCR MoM: OR 5.39; *p* = 0.001), and the prolonged pregnancy cohort (CPR MoM: OR 0.02; *p* = 0.01; UCR MoM: OR 15.02; *p* = 0.01). However, the results revealed no association between either ratio and emergency CD in SGA fetuses at term (CPR MoM: OR 0.64; *p* = 0.76; UCR MoM: OR 1.15; *p* = 0.86). Regarding the predictive value, a comparison of both ratios showed identical AUC. Regarding the Brier score, the UCR was slightly superior to the CPR at predicting emergency CD in the “Low Risk cohort” and in the “prolonged pregnancy cohort”. In contrast, the CPR was more predictive in the “Total cohort” and the “SGA cohort” (Table 3).

### 3.2. Operative Intervention

In SGA fetuses, the UCR was significantly associated with OI (OR 2.30; *p* = 0.01), while the CPR showed no association with OI (OR 0.42; *p* = 0.14). However, both the CPR and UCR were not associated with OI in the “low risk cohort” (CPR MoM: OR 1.01; *p* = 0.96; UCR MoM: OR 1.05; *p* = 0.73), the “prolonged pregnancy cohort” (CPR MoM: OR 0.84; *p* = 0.56; UCR MoM: OR 1.42; *p* = 0.24), or the “total cohort” (CPR MoM: OR 0.93; *p* = 0.57; UCR MoM: OR 1.24; *p* = 0.09). Regarding the prediction of operative intervention, the UCR was superior to the CPR in all the studied cohorts (Table 3).

### 3.3. Operative Intervention Due to Fetal Distress

Overall, our results showed a significant association between CPR MoM, UCR MoM, and OI due to fetal distress (CPR MoM: OR 0.50; *p* = 0.001; UCR MoM: OR 2.20; *p* ≤ 0.001). Regarding the low-risk cohort, the same results were demonstrated (CPR MoM: OR 0.61; *p* = 0.02; UCR MoM: OR 1.72; *p* = 0.009). However, in SGA fetuses, we only identified a significant association between the UCR MoM and OI due to fetal distress (OR 2.61; *p* = 0.008), while the CPR MoM was not associated with this outcome parameter (CPR MoM: OR 0.40; *p* = 0.20). In prolonged pregnancies, our results did not demonstrate any association between Doppler parameters and OI due to fetal distress (CPR MoM: OR 0.63; *p* = 0.28; UCR MoM: OR 1.69; *p* = 0.20). Similar to the outcome parameter “operative intervention,” the UCR was superior to the CPR at predicting operative intervention in all the examined cohorts due to fetal distress (Table 3). 

### 3.4. Five-Minute Apgar < 7

In the total cohort, the results showed a significant association between the UCR MoM and 5-min Apgar < 7 (OR 2.05; *p* = 0.04). However, there was no association with the CPR MoM (OR 0.43; *p* = 0.05). The analysis of subgroups revealed no associations between either ratio and 5-min Apgar < 7, neither in the low-risk cohort (CPR MoM: OR 0.49; *p* = 0.13; UCR MoM: OR 2.02; *p* = 0.10), nor in the prolonged pregnancy cohort (CPR MoM: OR 0.68; *p* = 0.64; UCR MoM: OR 2.13; *p* = 0.32), nor in SGA fetuses (CPR MoM: OR 0.31; *p* = 0.48; UCR MoM: OR 1.29; *p* = 0.77). While the CPR was superior to the UCR in the “total cohort”, the prediction using UCR was better than the CPR in the studied subgroups (Table 3). 

### 3.5. NICU Admission

Our results demonstrated an association between NICU admission, the UCR MoM (OR 2.31; *p* ≤ 0.001), and the CPR in the total cohort (OR 0.58; *p* = 0.002). Furthermore, a strong association was shown in SGA fetuses, both with the CPR and UCR (CPR MoM: OR 0.10; *p* ≤ 0.001; UCR MoM: OR 5.58; *p* ≤ 0.001). However, neither the CPR nor the UCR was associated with NICU admission in low-risk fetuses (CPR MoM: OR 0.88; *p* = 0.49; UCR MoM: OR 1.39; *p* = 0.08) or prolonged pregnancies (CPR MoM: OR 0.76; *p* = 0.52; UCR MoM: OR 1.39; *p* = 0.42). For all the studied cohorts, the prediction of NICU was improved using the UCR compared to the CPR (Table 3).

### 3.6. Combined Adverse Perinatal Outcome

There was a significant association between both the CPR and UCR with CAPO in the total cohort (CPR MoM: OR 0.59; *p* = 0.001; UCR MoM: OR 2.19; *p* ≤ 0.001). Furthermore, CAPO was associated with the CPR and UCR (CPR MoM: OR 0.12; *p* = 0.001; UCR MoM: OR 5.06; *p* ≤ 0.001) in SGA fetuses. While there was no association in prolonged pregnancies (CPR MoM: OR 0.61; *p* = 0.21; UCR MoM: OR 1.71; *p* = 0.15), the UCR (OR 1.42; *p* = 0.048), but not the CPR (OR 0.84; *p* = 0.30), was related to CAPO in the low-risk fetuses at term. The UCR was also superior to the CPR at predicting CAPO in all the investigated cohorts (Table 3).

## 4. Discussion

Many studies have used the CPR to predict adverse perinatal outcomes over the past years. A secondary analysis of the TRUFFLE study revealed that the 2-year outcome in fetuses with early growth restriction was better associated with the UCR than with the CPR. Since then, there has been ongoing international debate regarding the potential of reversing the ratio to the UCR for improving the prediction of even various short-term outcome parameters. The studies published on this topic are similar because they focus on specific cohorts, partly with small numbers of cases. This study complements the existing evidence on this topic in different settings. Regarding “small babies”, a study retrospectively examined both the UCR and the CPR with regard to the prediction of different outcome parameters in 172 SGA fetuses and 161 FGR fetuses. There was evidence that the UCR had a stronger association with almost all outcome parameters than the CPR. Nevertheless, there was no difference in the predictive value for an adverse outcome between the two ratios [21]. In our study, the UCR had a slight advantage over the CPR in predicting various outcome parameters. This was evident from the slightly lower Brier score in our statistical analysis. Nevertheless, the results of our study show that the difference in the Brier score is too small to conclude that the UCR is superior to the CPR. However, one difference is that our cohort includes all fetuses with birth weight < the 10th centile. We did not distinguish between FGR and SGA fetuses due to the retrospective study design. In this regard, our study is not a methodological exception: in a secondary analysis of a prospective study from the US, the authors included all fetuses with a sonographic-estimated fetal weight < the 10th centile and compared the predictive value of the CPR and UCR without differentiating between SGA and FGR fetuses. Again, in this population, there was no difference between either ratio in terms of predicting a CAPO [22]. 

The evidence regarding the “CPR versus UCR” in low-risk collectives—in contrast to neonates with birth weight < the 10th centile—has been limited so far, but has now been extended by our study. In term (AGA) fetuses with delivery from 37 weeks’ gestation, we were able to show an association between both ratios and emergency cesarean delivery and operative intervention due to fetal distress. For the CPR, this is consistent with the results of a prospective study on 775 fetuses. That study revealed that fetuses with a low CPR (<0.6765 MoM) had a significantly higher risk of emergency cesarean delivery or abnormal CTG compared to fetuses with a CPR ≥ 0.6765 MoM [23]. However, the potential for the UCR to be more predictive of these adverse outcomes in clinical practice—due to its marginally better calculation—remains to be debated. It seems reasonable to assume that the UCR does not have a large benefit in identifying fetuses at increased risk for an adverse outcome. Similarly, this is consistent with the findings of an Italian study comparing the UCR and CPR in a low-risk setting. The authors of that study could not find any difference in terms of prediction. Hence, they were not able to recommend the use of a specific ratio [24]. In prolonged pregnancies, the available evidence for both ratios has been the most limited to date. The present study demonstrated a significant association between the CPR and UCR with the need for emergency cesarean delivery. However, a significant improvement in the prediction of adverse perinatal outcomes could not be demonstrated using the UCR. Regarding the CPR, the results are consistent with an earlier study from 1989, which demonstrated that the CPR was significantly lower in cases with emergency cesarean delivery compared with fetuses with favorable outcomes. Ortiz et al. recently demonstrated that a low CPR was associated with a higher risk of operative delivery in fetuses with prolonged pregnancies [25]. However—in addition to emergency cesarean delivery—our study could not reveal any association between operative intervention and Doppler ratios or between the CPR and UCR. However, comparing our results with the study by Ortiz is challenging as the statistical approach used in both studies is different. While our study investigated the association between Doppler ratios and several outcome parameters, Ortiz et al. studied fetuses with a CPR < 10. centile versus fetuses with a “normal” CPR. Therefore, the results of both studies are only comparable to a limited extent. Thus, the data on prolonged pregnancies remain heterogeneous even in more contemporary studies. To our knowledge, the UCR has not been previously examined in prolonged pregnancies. However, our results suggest that reversing the ratio does not add much benefit to these fetuses. 

Summarizing the results from all investigated subgroups, it is concluded that the UCR does not significantly improve the prediction of adverse perinatal outcomes. Hence, we can confirm published studies on this topic in a large number of cases in different collectives.

As the reversal of the CPR to UCR does not show a significant benefit in predicting an adverse outcome, other approaches should be increasingly considered. For example, a 2017 study by Sirico et al. [26] showed that the CPR adjusted for fetal estimated weight was significantly associated with the presence of a pathological CTG and a low Apgar score. In later pregnancy (≥34 weeks’ gestation), the adjusted CPR was also associated with low birth pH (≤7.10) and meconium-stained amniotic fluid. In addition, the authors demonstrated that fetal estimated weight showed a significant positive correlation with the CPR MoM after linear regression. The group of Morales-Rosellò also showed that a fetus with low birth weight had a significantly lower CPR MoM [14]. Based on the data shown, future studies should also focus on adjusting the CPR for fetal estimated weight. 

### 4.1. Strengths of This Study

The strength of our study is the high number of cases. It is certainly likely that there are studies with a higher number of cases in the low-risk collective. However, in the prolonged pregnancy cohort, we provided new evidence on a very high number of cases.

### 4.2. Limitations

A limitation of our study is the retrospective analysis. Due to the design, the data are not blinded to the obstetrician; however, due to a lack of general recommendations on the appropriate use of the CPR, it is not considered in a decision-making process in clinical routine anyway. Furthermore, the authors would like to point out that perinatal and neonatal outcomes are subject to multifactorial factors and that, although the Doppler parameters studied may provide an insight, other factors should not be overlooked.

## 5. Conclusions

Our study provides further evidence comparing the CPR vs. UCR in different subgroups. The results of our study suggest that the reversal of the CPR to UCR does not improve the prediction of adverse perinatal outcomes.

## Figures and Tables

**Table 1:** Descriptive data for the different study cohorts. CAPO, combined adverse perinatal outcome; SGA, small-for-gestational-age.

	All Deliveries	Emergency Cesarean Delivery	Operative Intervention	Operative Intervention Due to Fetal Distress	5-Min Apgar < 7	NICU Admission	CAPO
Number							
Total cohort	3326 (100%)	37 (1.11%)	851 (25.59%)	320 (9.92%)	67 (2.08%)	437 (13.14%)	504 (15.15%)
Low-risk cohort	3105 (100%)	30 (0.97%)	783 (25.22%)	280 (9.02%)	59 (1.9%)	369 (11.88%)	432 (13.91%)
Prolonged pregnancy cohort	685 (100%)	8 (1.17%)	199 (29.10%)	82 (11.97%)	20 (2.92%)	81 (11.82%)	100 (14.60%)
SGA cohort	212 (100%)	7 (3.3%)	65 (30.66%)	38 (17.92%)	6 (2.83%)	62 (29.25%)	66 (31.13%)
Maternal age (years, median)							
Total cohort	32 (16–47)	32 (16–47)	32 (16–47)	32 (16–47)	32 (19–42)	32 (19–46)	32 (19–47)
Low-risk cohort	32 (16–47)	32 (16–47)	32 (16–47)	32 (16–47)	32 (19–42)	32 (19–46)	32 (19–47)
Prolonged pregnancy cohort	32 (16–47)	35.5 (29–47)	33 (16–47)	32 (19–47)	34 (22–38)	32 (19–42)	32 (19–47)
SGA cohort	31 (18–44)	32 (26–40)	31 (20–41)	31 (25–40)	32 (20–37)	31 (20–41)	31.5 (20–41)
Gravida (median, min–max)							
Total cohort	2 (1–12)	1 (1–9)	1 (1–12)	1(1–9)	1 (1–9)	2 (1–9)	2 (1–9)
Low-risk cohort	2 (1–12)	1 (1–9)	1 (1–12)	1 (1–9)	1 (1–9)	2 (1–9)	2 (1–9)
Prolonged pregnancy cohort	2 (1–9)	1(1–3)	1 (1–5)	1 (1–4)	1.5 (1–3)	1 (1–9)	1 (1–9)
SGA cohort	1 (1–9)	1 (1–3)	1 (1–5)	1 (1–5)	1 (1–2)	1 (1–6)	1 (1–6)
Para (median, min–max)							
Total cohort	1 (0–9)	1 (1–6)	1 (0–6)	1 (0–6)	1 (1–6)	1 (0–9)	1 (0–9)
Low-risk cohort	1 (0–9)	1 (1–6)	1 (0–6)	1 (0–6)	1 (1–6)	1 (1–9)	1 (1–9)
Prolonged pregnancy cohort	1 (0–9)	1 (1–2)	1 (1–3)	1 (1–3)	1 (1–3)	1 (1–9)	1 (1–9)
SGA cohort	1 (0–6)	1 (1–6)	1 (1–3)	1 (1–3)	1 (1–2)	1 (0–3)	1 (0–3)
Maternal BMI (median, min–max)							
Total cohort	28.5 (18.5–60.6)	29.3 (21.6–47.4)	29 (19.5–59)	29.1 (20.5–59)	28.9 (21.3–52.8)	28.9 (19–59)	29 (19–59)
Low-risk cohort	28.6 (18.5–59)	30.1 (24.6–47.4)	29.1 (19.5–59)	29.1 (20.5–59)	29 (21.3–52.8)	29.1 (19–59)	29.1 (19–59)
Prolonged pregnancy cohort	28.4 (20.6–52.8)	32.1 (26.2–36.3)	28.9 (23.1–52.8)	28.8 (23.1–38.8)	31.2 (21.3–52.8)	30.8 (21.1–40.9)	30.7 (21.1–52.8)
SGA cohort	27.8 (20.1–60.6)	27.8 (21.6–32.4)	28.2 (21.6–43.8)	28.4 (21.6–43.8)	27.4 (21.6–39.7)	27.7 (21.6–43.8)	27.7 (21.6–43.7)
Ethnicity: number (%)							
Europe							
Total cohort	1757 (52.83%)	19 (51.14%)	443 (52.06%)	163 (50.94%)	30 (44.78%)	209 (47.83%)	243 (48.21%)
Low-risk cohort	1648 (53.08%)	16 (53.33%)	415 (53%)	145 (51.79%)	28 (47.46%)	178 (48.24%)	210 (48.61%)
Prolonged pregnancy cohort	389 (56.79%)	5 (62.5%)	109 (54.77%)	49 (59.76%)	10 (50%)	39 (48.15%)	49 (49%)
SGA cohort	106 (50%)	3 (42.85%)	28 (43.08%)	18 (47.37%)	2 (33.33%)	29 (46.77%)	31 (46.97%)
Others							
Total cohort	1569 (47.17%)	18 (48.6%)	408 (47.94%)	157 (49.06%)	37 (55.22%)	228 (52.17%)	261 (51.79%)
Low-risk cohort	1457 (46.92%)	14 (46.67%)	368 (47%)	135 (48.21%)	31 (34.83%)	191 (51.76%)	222 (51.39%)
Prolonged pregnancy cohort	296 (43.21%)	3 (37.5%)	90 (45.23%)	33 (40.24%)	10 (50%)	42 (51.85%)	51 (51%)
SGA cohort	106 (50%)	4 (57.14%)	37 (56.92%)	20 (52.63%)	4 (66.66%)	33 (53.23)	35 (53.03%)
Gestational diabetes: number (%)							
Total cohort	329 (9.89%)	7 (18.92%)	109 (12.81%)	39 (12.19%)	11 (16.42%)	68 (15.56%)	73 (14.48%)
Low-risk cohort	314 (10.11%)	6 (20%)	104 (13.28%)	35 (12.5%)	11 (18.64%)	65 (17.62%)	40 (16.20%)
Prolonged pregnancy cohort	40 (5.84%)	0 (0%)	18 (9.05%)	5 (6.10%)	2 (10%)	7 (8.64%)	9 (9%)
SGA cohort	15 (7.08%)	1 (14.29%)	5 (7.69%)	4 (10.53%)	0 (0%)	3 (4.84%)	3 (4.55%)
Hypertensive pregnancy disorders: number (%)							
Total cohort	88 (2.65%)	1 (2.70%)	30 (3.53%)	10 (3.16%)	7 (10.45%)	19 (4.35%)	22 (4.37%)
Low-risk cohort	76 (2.45%)	1 (3.33%)	26 (3.32%)	9 (3.21%)	7 (11.86%)	15 (4.07%)	18 (4.17%)
Prolonged pregnancy cohort	7 (1.02%)	1 (12.5%)	3 (1.51%)	2 (2.45%)	2 (10%)	2 (2.47%)	3 (3%)
SGA cohort	11 (5.19%)	0 (0%)	4 (6.15%)	1 (2.63%)	0 (0%)	4 (6.45%)	4 (6.06%)
Previous cesarean delivery: number (%)							
Total cohort	444 (13.35%)	8 (21.62%)	224 (26.32%)	57 (17.81%)	12 (17.91%)	56 (12.81%)	66 (13.10%)
Low-risk cohort	425 (13.69%)	7 (23.33%)	212 (27.08%)	50 (17.86%)	9 (15.25%)	48 (13.01%)	58 (13.43%)
Prolonged pregnancy cohort	76 (11.09%)	2 (25%)	38 (19.1%)	9 (10.78%)	3 (15%)	8 (9.88%)	12 (12%)
SGA cohort	17 (8.02%)	1 (14.29%)	10 (15.38%)	6 (15.79%)	1 (16.66%)	6 (9.68%)	6 (9.09%)
Induction of labor: number (%)							
Total cohort	1434 (43.11%)	20 (54.05%)	400 (47%)	167 (52.19%)	37 (55.22%)	230 (52.63%)	261 (51.79%)
Low-risk cohort	1284 (41.35%)	16 (53.33%)	356 (45.47%)	50 (17.86%)	33 (55.93%)	175 (47.43%)	203 (46.99%)
Prolonged pregnancy cohort	453 (66.13%)	8 (100%)	140 (70.35%)	59 (71.95%)	17 (85%)	60 (74.07%)	72 (72%)
SGA cohort	143 (67.45%)	4 (57.14%)	43 (66.15%)	30 (78.95%)	4 (66.66%)	51 (82.26%)	54 (81.81%)
Pathological CTG: number (%)							
Total cohort	681 (20.48%)	33 (89.19%)	320 (37.6%)	320 (100%)	30 (44.78%)	137 (31.35%)	169 (33.53%)
Low-risk cohort	616 (19.84%)	28 (93.33%)	280 (35.76%)	280 (100%)	27 (45.76%)	106 (28.73%)	137 (31.71%)
Prolonged pregnancy cohort	152 (22.19%)	8 (100%)	82 (41.21%)	82 (100%)	7 (35%)	25 (30.86%)	34 (34%)
SGA cohort	63 (29.72%)	5 (71.43%)	38 (58.46%)	38 (100%)	2 (33.33%)	29 (46.77%)	30 (45.45%)
GA at scan: weeks, median							
Total cohort	39.9 (35.1–42.7)	40 (36.7–41.3)	40 (35–42.3)	40.1 (35.5–42.1)	40.1 (35.6–41.4)	39.7 (35.1–42)	39.9 (35.1–42)
Low-risk cohort	40 (35.1–42.7)	40 (36.7–41.3)	40 (35.1–42.3)	40.1 (35.6–42.1)	40.1 (36.7–41.4)	40 (35.1–42)	40 (35–42)
Prolonged pregnancy cohort	41 (39.1–42.7)	41 (40.5–41.3)	41 (39.1–42.3)	41 (40–42)	41 (40.3–41.4)	41 (39.4–42)	41 (39.4–42)
SGA cohort	39.2 (37.0–42.1)	39.3 (37.3–41.3)	39.3 (37–42.1)	39.7 (37–42.4)	39.7 (37.1–41)	38 (37–41.4)	38.1 (37–42.4)
Interval scan-to-delivery: (days, median)							
Total cohort	1 (0–14)	2 (0–11)	1 (0–14)	1 (0–12)	1 (0–11)	1 (0–13)	1 (0–13)
Low-risk cohort	1 (0–14)	2 (0–11)	1 (0–14)	1 (0–12)	1 (0–10)	1 (0–13)	1 (0–13)
Prolonged pregnancy cohort	2 (0–14)	2.5 (2–4)	2 (0–14)	2 (0–7)	2 (0–6)	2 (0–11)	2 (0–11)
SGA cohort	1 (0–12)	1 (0–3)	1 (0–11)	1 (0–4)	1 (0–3)	2 (0–5)	1 (0–5)

**Table 2:** Univariate regression analysis results for the CPR and the UCR. *OR*, odds ratio; *CI*, confidence interval; *CAPO*, combined adverse perinatal outcome.

	CPR		UCR	
	AUC	Brier Score	AUC	Brier Score
Emergency cesarean delivery				
Total cohort	0.642	0.01096	0.642	0.01098
Low-risk cohort	0.643	0.00953	0.643	0.00952
Prolonged pregnancy cohort	0.752	0.01135	0.752	0.01119
SGA cohort	0.532	0.03192	0.532	0.03193
Operative intervention				
Total cohort	0.512	0.19038	0.512	0.19022
Low-risk cohort	0.507	0.18858	0.507	0.18857
Prolonged pregnancy cohort	0.517	0.20600	0.517	0.20565
SGA cohort	0.549	0.21012	0.549	0.20526
Operative intervention CTG				
Total cohort	0.555	0.08663	0.555	0.08618
Low-risk cohort	0.544	0.08190	0.544	0.08185
Prolonged pregnancy cohort	0.532	0.10519	0.532	0.10506
SGA cohort	0.558	0.14547	0.558	0.13952
5-Minute Apgar < 7				
Total cohort	0.566	0.01971	0.566	0.01972
Low-risk cohort	0.556	0.01863	0.566	0.01862
Prolonged pregnancy cohort	0.520	0.02833	0.520	0.02826
SGA cohort	0.436	0.02747	0.436	0.02749
NICU admission				
Total cohort	0.547	0.11373	0.547	0.11266
Low-risk cohort	0.516	0.10470	0.516	0.10459
Prolonged pregnancy cohort	0.522	0.10420	0.522	0.10416
SGA cohort	0.647	0.19191	0.647	0.18313
CAPO				
Total cohort	0.544	0.12810	0.544	0.12708
Low-risk cohort	0.517	0.11973	0.517	0.11960
Prolonged pregnancy cohort	0.534	0.12438	0.534	0.12426
SGA cohort	0.638	0.20049	0.638	0.19244

**Table 3:** Univariate regression analysis results for the CPR and the UCR. *OR*; odds ratio; *CI*, confidence interval; *CAPO*, combined adverse perinatal outcome; *SGA*, small-for-gestational-age; *NICU*, neonatal intensive care unit; *CPR*, cerebroplacental ratio; *UCR*, umbilicocerebral ratio.

	CPR				UCR			
	Outcome	No Outcome	Univariate Analysis: OR (CI)	*p*-Value	Outcome	No Outcome	Univariate Analysis: OR (CI)	*p*-Value
Emergency cesarean delivery								
Total cohort	0.89	1.04	0.11 (0.03–0.42)	**0.001**	1.23	1.04	3.82 (1.81–7.30)	**0.001**
Low-risk cohort	0.90	1.06	0.10 (0.02–0.45)	**0.002**	1.22	1.02	5.39 (2.10–12.39)	**0.001**
Prolonged pregancy cohort	0.81	1.04	0.02 (0.00–0.40)	**0.01**	1.34	1.03	15.02 (2.24–89.09)	**0.01**
SGA cohort	0.85	0.88	0.64 (0.03–10.72)	0.76	1.28	1.25	1.15 (0.16–4.19)	0.86
Operative intervention								
Total cohort	1.04	1.04	0.93 (0.71–1.20)	0.57	1.05	1.03	1.24 (0.97–1.59)	0.09
Low-risk cohort	1.06	1.05	1.01 (0.77–1.32)	0.96	1.03	1.02	1.05 (0.79–1.39)	0.73
Prolonged pregancy cohort	1.03	1.04	0.84 (0.46–1.51)	0.56	1.05	1.02	1.42 (0.79–2.52)	0.24
SGA cohort	0.84	0.90	0.42 (0.13–1.32)	0.14	1.37	1.20	2.30 (1.21–4.64)	**0.01**
Operative intervention CTG								
Total cohort	0.99	1.05	0.50 (0.33–0.76)	**0.001**	1.12	1.03	2.20 (1.57–3.06)	**<0.001**
Low-risk cohort	1.02	1.06	0.61 (0.39–0.93)	**0.02**	1.07	1.02	1.72 (1.14–2.55)	**0.009**
Prolonged pregancy cohort	1.01	1.04	0.63 (0.26–1.44)	0.28	1.07	1.03	1.69 (0.76–3.64)	0.20
SGA cohort	0.83	0.89	0.40 (0.09–1.59)	0.20	1.44	1.21	2.61 (1.29–5.52)	**0.008**
5-min Apgar < 7								
Total cohort	0.98	1.04	0.43 (0.17–1.01)	0.05	1.12	1.04	2.05 (1.02–3.77)	**0.04**
Low-risk cohort	1.00	1.06	0.49 (0.19–1.22)	0.13	1.09	1.02	2.02 (0.88–4.31)	0.10
Prolonged pregancy cohort	1.01	1.04	0.68 (0.12–3.22)	0.64	1.10	1.03	2.13 (0.46–8.36)	0.32
SGA cohort	0.81	0.88	0.31 (0.001–7.14)	0.48	1.31	1.25	1.29 (0.17–4.81)	0.77
NICU admission								
Total cohort	1.00	1.05	0.58 (0.41–0.83)	**0.002**	1.12	1.03	2.31 (1.71–3.10)	**<0.001**
Low-risk cohort	1.04	1.06	0.88 (0.61–1.26)	0.49	1.05	1.02	1.39 (0.96–2.00)	0.08
Prolonged pregancy cohort	1.02	1.04	0.76 (0.32–1.73)	0.52	1.06	1.03	1.39 (0.61–3.04)	0.42
SGA cohort	0.78	0.92	0.10 (0.003–0.37)	**<0.001**	1.48	1.16	5.58 (2.59–13.20)	**<0.001**
CAPO								
Total cohort	1.00	1.05	0.59 (0.42–0.82)	**0.001**	1.11	1.03	2.19 (1.64–2.9)	**<0.001**
Low-risk cohort	1.04	1.06	0.84 (0.59–1.18)	0.30	1.05	1.02	1.42 (1.00–1.98)	**0.048**
Prolonged pregancy cohort	1.01	1.05	0.61 (0.27–1.31)	0.21	1.07	1.03	1.71 (0.82–3.48)	0.15
SGA cohort	0.79	0.92	0.12 (0.03–0.41)	**0.001**	1.46	1.16	5.06 (2.39–11.75)	**<0.001**

## Data Availability

Not applicable.

## Appendix A

**Table A1:** ROC analyses and Brier scores for UA and MCA. *OR*, odds ratio; *CI*, confidence interval; *CAPO*, combined adverse perinatal outcome; *UA*, umbilical artery; *MCA*, middle cerebral artery.

	UA		MCA	
	AUC	Brier Score	AUC	Brier Score
Emergency cesarean delivery				
Total cohort	0.606	0.01099	0.610	0.01097
Low-risk cohort	0.616	0.00956	0.599	0.00955
Prolonged pregnancy cohort	0.660	0.01150	0.662	0.01145
SGA cohort	0.544	0.03187	0.594	0.03179
Operative intervention				
Total cohort	0.515	0.19021	0.500	0.19039
Low-risk cohort	0.513	0.18851	0.507	0.18853
Prolonged pregnancy cohort	0.511	0.20592	0.512	0.20609
SGA cohort	0.511	0.21155	0.580	0.20825
Operative intervention CTG				
Total cohort	0.550	0.08647	0.529	0.08688
Low-risk cohort	0.546	0.08187	0.514	0.08203
Prolonged pregnancy cohort	0.532	0.10508	0.515	0.10536
SGA cohort	0.514	0.14601	0.610	0.14308
5-min Apgar < 7				
Total cohort	0.524	0.01973	0.548	0.01972
Low-risk cohort	0.498	0.01864	0.559	0.01862
Prolonged pregnancy cohort	0.534	0.02834	0.562	0.02829
SGA cohort	0.627	0.02750	0.550	0.02750
NICU admission				
Total cohort	0.512	0.11394	0.559	0.11367
Low-risk cohort	0.520	0.10467	0.544	0.10454
Prolonged pregnancy cohort	0.527	0.10416	0.561	0.10394
SGA cohort	0.623	0.19598	0.619	0.20040
CAPO				
Total cohort	0.516	0.12834	0.552	0.12810
Low-risk cohort	0.511	0.11976	0.538	0.11958
Prolonged pregnancy cohort	0.502	0.12467	0.554	0.12425
SGA cohort	0.612	0.20479	0.613	0.20781

**Table A2:** Univariate regression analyses for UA PI MoM and MCA PI MoM. *OR*, odds ratio; *CI*, confidence interval; *CAPO*, combined adverse perinatal outcome; *UA*, umbilical artery; *MCA*, middle cerebral artery; *PI*, pulsatility index; *MoM*, Multiple of Median; *SGA*, small-for-gestational-age; *NICU*, neonatal intensive care unit.

	UA PI MoM				MCA PI MoM			
	Outcome	No Outcome	Univariate Analysis: OR (CI)	*p*-Value	Outcome	No Outcome	Univariate Analysis: OR (CI)	*p*-Value
Emergency cesarean delivery								
Total cohort	1.08	1.02	3.87 (0.88–14.37)	0.07	0.94	1.04	0.13 (0.02–0.61)	**0.008**
Low-risk cohort	1.09	1.01	6.00 (1.07–28.35)	**0.04**	0.95	1.04	0.16 (0.03–0.87)	**0.03**
Prolonged pregancy cohort	1.12	1.01	15.91 (0.44–536.27)	0.13	0.88	1.03	0.03 (0.00–1.01)	0.05
SGA cohort	1.07	1.14	0.30 (0.01–5.88)	0.47	0.88	0.96	0.11 (0.00–4.94)	0.28
Operative intervention								
Total cohort	1.03	1.02	1.42 (0.96–2.07)	0.08	1.04	1.03	1.05 (0.75–1.45)	0.78
Low-risk cohort	1.02	1.01	1.27 (0.83–1.93)	0.27	1.05	1.04	1.17 (0.83–1.63)	0.37
Prolonged pregancy cohort	1.02	1.01	1.44 (0.59–3.48)	0.42	1.02	1.03	0.90 (0.44–1.81)	0.76
SGA cohort	1.16	1.12	1.72 (0.56–5.32)	0.34	0.91	0.98	0.21 (0.04–0.91)	**0.04**
Operative intervention CTG								
Total cohort	1.06	1.02	2.89 (1.68–4.93)	**<0.001**	1.01	1.04	0.66 (0.40–1.08)	0.10
Low-risk cohort	1.04	1.01	2.30 (1.23–4.24)	**0.009**	1.03	1.04	0.83 (0.49–1.39)	0.49
Prolonged pregancy cohort	1.04	1.01	2.23 (0.65–7.51)	0.20	1.02	1.03	0.84 (0.3–2.23)	0.72
SGA cohort	1.17	1.13	1.94 (0.50–7.01)	0.33	0.89	0.97	0.12 (0.02–0.78)	**0.03**
5-min Apgar < 7								
Total cohort	1.04	1.02	1.68 (0.51–5.09)	0.39	0.98	1.04	0.36 (0.11–1.05)	0.06
Low-risk cohort	1.01	1.01	1.02 (0.26–3.77)	0.98	0.98	1.04	0.33 (0.10–1.02)	0.06
Prolonged pregancy cohort	0.99	1.01	0.57 (0.05–6.14)	0.65	0.97	1.03	0.31 (0.03–2.25)	0.26
SGA cohort	1.18	1.13	1.97 (0.07–23.53)	0.65	0.95	0.96	0.87 (0.01–27.19)	0.95
NICU admission								
Total cohort	1.04	1.02	1.67 (1.02–2.70)	**0.04**	1.00	1.04	0.47 (0.30–0.73)	**0.001**
Low-risk cohort	1.00	1.01	0.71 (0.50–1.26)	0.25	1.01	1.04	0.60 (0.37–0.96)	**0.03**
Prolonged pregancy cohort	1.00	1.02	0.57 (0.16–1.99)	0.38	0.99	1.03	0.49 (0.17–1.36)	0.18
SGA cohort	1.22	1.10	6.91 (2.13–24.47)	**0.001**	0.91	0.98	0.17 (0.03–0.77)	**0.02**
CAPO								
Total cohort	1.04	1.02	1.68 (1.06–2.65)	**0.03**	1.00	1.04	0.50 (0.33–0.76)	**0.001**
Low-risk cohort	1.01	1.01	0.84 (0.49–1.42)	0.51	1.02	1.04	0.62 (0.40–0.96)	**0.03**
Prolonged pregancy cohort	1.01	1.01	0.98 (0.31–3.05)	0.98	1.00	1.03	0.50 (0.19–1.26)	0.14
SGA cohort	1.21	1.10	5.85 (1.85–20.08)	**0.002**	0.91	0.98	0.18 (0.04–0.76)	**0.02**
